# Density functional theory and molecular dynamics simulation-based bioprospection of *Agathosma betulina* essential oil metabolites against protein tyrosine phosphatase 1B for interventive antidiabetic therapy

**DOI:** 10.1016/j.heliyon.2025.e42239

**Published:** 2025-01-24

**Authors:** Oluwaseye Adedirin, Rukayat A. Abdulsalam, Khadeejah O. Nasir-Naeem, Ayenitaju A. Oke, Akolade O. Jubril, Saheed Sabiu

**Affiliations:** a Department of Biotechnology and Food Science, Faculty of Applied Sciences, Durban University of Technology, PO Box 1334, Durban, 4000, South Africa; b Biotechnology Advanced Research Centre, Sheda Science and Technology Complex, PO Box 186, Garki, Abuja, Nigeria

**Keywords:** Diabetes, Buchu, Essential oil, PTP1B, Computational screening

## Abstract

Type II diabetes mellitus (T2DM) is characterized by elevated blood glucose due to impaired insulin secretion/sensitivity. While conventional antihyperglycemic medications like biguanides, sulfonylureas, and other agents are commonly used, their long-term use can have side effects, prompting research into natural alternatives. This study bioprospects the antidiabetic potential of metabolites in *Agathosma betulina* (Buchu) essential oil through computational analysis of their ability to inhibit protein tyrosine phosphatase 1B (PTP1B), a therapeutic diabetes target. Molecular dynamic simulation, supported by DFT analysis, revealed that compounds linalylanthranilate (−20.18 kcal/mol) and γ-diosphenol (−16.49 kcal/mol) found in the oil exhibited stronger PTP1B inhibition than ursolic acid (−15.98 kcal/mol). The compounds showed favorable drug-like properties complying with Lipinski's rules. This study provides the first evidence that these Buchu oil compounds could potentially serve as PTP1B inhibitors to enhance insulin receptor sensitivity, showing promise for T2DM treatment. Further validation through safety and clinical studies is recommended.

## Highlights

• Exploring alternative therapies for anti-type II diabetes mellitus agents is ongoing.
• PTP1B inhibitors from Buchu oil were explored using computational methods.
• ΔG_bind_ of Linalylanthranilate, γ-diosphenol from buchu oil outperformed ursolic acid.
• DFT reactivity indices obtained corroborate observed ΔG_bind_ values.
• Obtained compounds may treat T2DM by inhibiting PTP1B and enhancing insulin sensitivity.

## Graphical abstract

**Figure:**
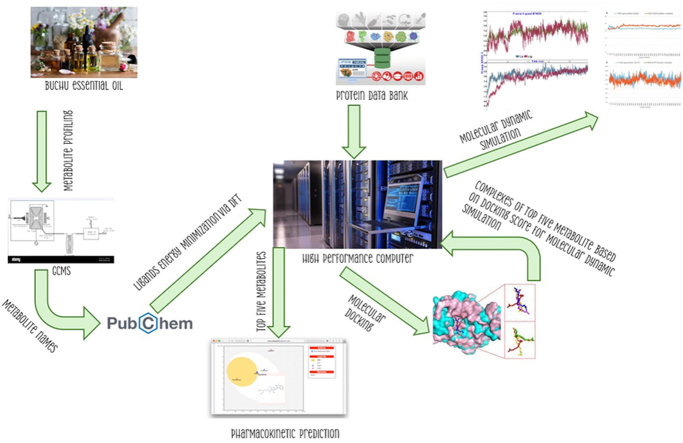

## 1 Introduction

Type II diabetes mellitus (T2DM) is a chronic disorder characterized by elevated blood sugar levels due to reduced insulin sensitivity or secretion, affecting nearly 450 million people globally with projections to double by 2030. It can lead to severe complications such as heart attacks, strokes, amputations, and kidney failure if left untreated [1]. While there is no outright cure, T2DM is typically managed with anti-hyperglycemic agents like biguanides, meglitinide, sulfonylureas, thiazolidinediones, GLP-1 inhibitors, alpha glucosidase inhibitors, dipeptidyl peptidase inhibitors, and SGT2 inhibitors [2]. However, the prolonged use of these medications can cause mild side effects, prompting the search for alternative treatments. Several proteins and enzymes have been implicated in T2DM disease including protein tyrosine phosphatase 1B (PTP1B). PTP1B plays a crucial role in insulin regulation by dephosphorylating tyrosine residues on insulin receptors (IR) and insulin receptor substrates (IRS), acting as a negative regulator [3]. Inhibition of PTP1B has been shown to increase insulin sensitivity and secretion, making it a promising target for T2DM therapy [4].

In recent years, there has been renewed interest in exploring medicinal plants for drug discovery and development [5]. This approach is favored for its accessibility, lower costs, and potentially fewer side effects. *Agathosma betulina*, commonly known as buchu in South Africa, is an aromatic plant traditionally used to treat various ailments, including T2DM, urinary tract infections, and hematuria [6,7]. Also, its leaves essential oil exhibits various biological activities, including antioxidantαand diureticαeffects [8]. Numerous studies have documented the inhibitory effects of essential oils on key enzymes implicated in T2DM; however, investigations regarding their potential inhibitory activity against PTP1B remain scarce in scientific literature [9,10]. Previous investigations demonstrated the inhibitory potential of pimelyl dihydrazide from *an* essential oil against PTP1B [11]. In another study, cinnamaldehyde, a constituent isolated from another essential oil, exhibited significant PTP1B inhibitory activity, as evidenced through both enzymatic assays and cell viability assessments [12]. The therapeutic efficacy of buchu essential oil in exhibiting antidiabetic properties, specifically against T2DM, is attributed to its inherent phytoconstituents [13]. Consequently, it is crucial to investigate the role of these phytoconstituents in the oil's antidiabetic effects, as well as to elucidate the molecular mechanisms underlying these effects, particularly in relation to the enzyme protein tyrosine phosphatase 1B (PTP1B). Therefore, this study aims to bioprospects buchu essential oil metabolites as potential PTP1B inhibitors for interventive diabetes treatment, using molecular docking, dynamics simulations, pharmacokinetics screening, and density functional theory techniques.

## 2 Material and methods

### 2.1 Ligand acquisition and preparation

Buchu essential oil was sourced from Soil Organics, SouthiAfrica and its metabolites was profiled using Shimadzu GCMS-QP2010SE gas chromatograph-mass spectrometer. The instrument separating column consisted of Shimadzu RTX-5 capillary column (30 m × 0.32 mm × 1.0 μm) and helium as the carrier gas at 1.0 mL/min rate. The electron-impact ionization method was used with electron energy of 70 eV and ion source temperature of 230 °C. The GC was operated with a split ratio of 5:1 and an injection port temperature of 250 °C. The oven temperature was adjusted from 60 °C for 1 min, increased to 120 °C at 2 min, and then increased at 5 °C per minute up to 290 °C (which was then held for 3min). The instrument identified the components of the buchu essential oil via selected ion monitoring (SIM) mode and by comparing their retention indices and mass spectra with those in the NIST147 libraries. Thereafter, the percentage compositions of the oil were computed from GC peak areas. The 3Distructures of the identified metabolites (ligands) were obtained from PubChem database (https://pubchem.ncbi.nlm.nih.gov/) in standard data format (SDF). Molecular geometry of the structures was optimized through DFT B3LYP/631G∗ quantum mechanical methods using Spartan 14 software [14]. Optimized structures were saved in protein data bank (PDB) format and subsequently converted to PDBQT format using Auto Dock Tool [15].

### 2.2 Protein target preparation

The protein target in the study was human protein tyrosine phosphatase 1B (PTP1B) co-crystallized with amorphadiene (PDB:6W30) obtained from RCSB database (https://www.rcsb.org). The x-ray crystal structure was without mutation and had 2.10 Å resolution. Using Discovery Studio 2021 client [16], the amorphadiene binding site was identified at coordinates 37.49: −32.56: 19.17 Å. After extracting the co-crystallized ligand using PyMol [17], the protein structure was optimized using Auto Dock Tools. The relics of x-ray crystallization were removed including water and other heteroatoms, polar hydrogen atoms were added, and the atoms were assigned AD4-type atoms. Kollman charges and Gasteiger charges were also computed. The final structure was saved in PDBQT format for docking study.

### 2.3 Molecular docking study

The molecular docking analysis was conducted using Auto Dock Vina. The protocol for docking study was established by redocking the extracted co-crystallized ligand into the protein's binding site (coordinates: 37.49, −32.56, 19.17 Å). The grid box radius was iteratively adjusted until the docked pose achieved an RMSD value below 1 when compared to the experimental crystallographic pose obtained from RCSB PDB database. Discovery Studio Client 2021 was used for poses comparison and RMSD calculations. Thereafter, the optimized protocol was employed to dock the ligands against the binding domain of PTP1B. Ursolic acid (validated inhibitor of PTP1B) [18] was used as reference molecule in the study. The ligands were ranked based on their docking score and the number of interactions with the target molecules. Consequently, the top five ligands were selected for molecular dynamic studies.

### 2.4 Molecular dynamics simulation studies

Molecular dynamics (MD) simulations were performed using AMBER 18 with the FF18SB force field for 150 ns, following previously established protocols [19]. Ligand atomic partial charges were generated using ANTECHAMBER with general amber force field (GAFF) parameters and constrained electrostatic potential (RESP) calculations. The protein-ligand systems containing 287 amino acid sequence were neutralized using hydrogen atoms, Na^+^ and Cl^−^ counter ions via the Leap module. Each system was solvated in an orthorhombic TIP3P water box with an 8 Å buffer distance. The SHAKE algorithm was employed to constrain all hydrogen atom bonds. Simulations were conducted using a 2 fs time step under isobaric-isothermal (NPT) conditions, with randomized seeding at 300K and 1 bar pressure. Temperature and pressure control were maintained using a Langevin thermostat (collision frequency: 1.0 ps^−1^) and a pressure-coupling constant of 2 ps, respectively. The resulting 150 ns trajectories were analyzed as post-dynamic data. Complex interactions at 150 ns were visualized using Discovery Studio client 2021.

### 2.5 Post dynamic simulation analysis

Post-dynamic analysis was conducted according to established protocols [19]. Trajectory analysis was performed using the PTRAJ module of AMBER 18 to combine and evaluate simulated coordinates. The CPPTRAJ module was employed to calculate root-mean-square deviation (RMSD), root-mean-square fluctuation (RMSF), radius of gyration (ROG), and solvent-accessible surface area (SASA), with subsequent visualization using Origin v 6.0 as described by Sabiu et al. [20]. The relative free binding energy (ΔG) of the complexes analyzing 100,000 snapshots of the 150 ns trajectories were computed using thermal molecular mechanics generalized born surface area (MMGBSA) method MMGBSA method. Expressions for ΔG calculation were represented by equations (1), (2), (3), (4), (5)):

(1) $${\Delta G}_{bind}=\left(E_{gas}+G_{sol}\right)-TS$$

(2) $${\Delta G}_{bind}=E_{complex}-\left(G_{receptor}+G_{ligand}\right)$$

(3) $$E_{gas}=E_{\mathrm{int}}-E_{vdW}+G_{ele}$$

(4) $$G_{sol}=E_{GB}-G_{SA}$$

(5) $$G_{SA}=E_{\gamma SASA}$$

In equations (1), (2), (3), (4), (5)), E_gas_ is the gas-phase energy; E_int_ is the internal energy; E_ele_ is coulomb energy; E_vdw_ is the van der Waals energy; G_sol_ is solvation-free energy from polar state; GGB is the difference between solvation (G_SA_) and free energy from polar state non-polar states E_GB_; S is total entropy, and T is temperature in Kelvin.

### 2.6 Density function theory calculation

Additional information about the reactivity of the top five ligands was studied through Density function theory (DFT) calculation. Spartan 14 software was used for calculation. A combination of Merck Molecular Force Field (MMFF) [21] and Becke's three exchange functional-Lee-Yang-Parr gradient corrected correlation functional (B3LYP) [22,23]was used to optimize the equilibrium geometry of molecules using a 6-31G∗ basis set [24]. To ensure convergence, the number of unpaired electrons in the molecules was set to 0 and the optimization cycle was set to 1,000,000. Upon completion, quantum mechanical properties including lowest unoccupied molecular orbital energy (E_LUMO), highest occupied molecular orbital energy (E_HOMO), dipole moment (Debye) and polarizability were obtained from the software's property module. Other conceptual DFT properties (equations 6–13) were calculated from the obtained properties based on Parr and Pearson interpretation and Koopmans theorem [25,26].

(6) $$\text{Energy}\;\text{gap}\Delta E\;\left(\text{eV}\right)=E_{\text{LUMO}}-E_{\text{HOMO}}$$

(7) $$\text{First}\;\text{ionization}\;\text{energy}\;I\left(\text{eV}\right)=-E_{\text{HOMO}}$$

(8) $$\text{Electron}\;\text{affinity}\;A\;\left(\text{eV}\right)=-E_{\text{LUMO}}$$

(9) $$\text{Hardness}\;\eta \;\left(\text{eV}\right)=\frac{I-A}{2}$$

(10) $$\text{Softness}\;\varsigma \;\left(\text{eV}\right)=\frac{1}{\eta }$$

(11) $$\text{Electronegativity}\;\text{index}\;\chi \;\left(\text{eV}\right)=\frac{I+A}{2}$$

(12) Chemicalpotentialμ(eV)=−χ

(13) $$\text{Electrophilicity}\;\text{index}\;\omega =\frac{\mu^{2}}{2\eta }$$

### 2.7 Pharmacokinetic assessment

Pharmacokinetic and druglike properties of the top five ligands were studied using SwissADME webserver (http://www.swissadme.ch/index/php). Also, Protox II webserver (https://tox-new.charite.de/protox_II/) was utilized to evaluate their toxicity profile. Briefly, SDF format of the optimized ligand's structure was uploaded into SwissADME and subsequently converted to canonical SMILES format for the calculation. From the results obtained, Lipinski rule of five (Ro5) was used to Judge the compounds drug likeness. Similarly, the SMILES format of the compounds was uploaded to Protox II webserver for the toxicity profile of the compounds.

## 3 Results and discussion

### 3.1 Metabolite profile of buchu essential oil

The selected ion monitoring (SIM) mode chromatogram, along with comprehensive data for the metabolite profiling of buchu essential oil using a Shimadzu GCMS-QP2010SE spectrometer, is presented in Supplementary Fig. S1 and Table S1. The analysis identified 21 distinct compounds, including monoterpenes, monoterpenoids, siloxanes, aromatic esters, cyclic ether diketones, and ketones (Table 1). The relative abundance of these compounds ranged from 5.87 % for p-Mentha-8-thiol, trans to 2.90 % for 2,3-dimethyl-2-(3-oxobutyl) cyclohexanone (Table 1). Consequently, p-Mentha-8-thiol, trans may significantly contribute to the various properties exhibited by the oil. The metabolic profile of the Buchu specimens analyzed in this investigation demonstrated consistency with previously documented compounds reported in peer-reviewed literatures [6,7,13] and established phytochemical databases, including Dr. Duke's Phytochemical and Ethnobotanical Database (https://phytochem.nal.usda.gov/).

**Table 1:** Metabolites from buchu essential oil used in this study.

Name	R.T.	A/H	RA %	Class
Alpha-pinene	5.60	1.79	2.92	Monoterpene
Beta-cymene	6.74	3.59	5.85	Monoterpene
Eucalyptol	6.82	2.22	3.62	Monoterpenoid
L-Fenchone	7.52	1.80	2.93	Monoterpenoid
Beta-linalool	7.76	2.47	4.03	Acyclic monoterpenoid
Linalool, methyl ether	8.34	2.15	3.51	Acyclic monoterpenoid
P-menthone	8.52	3.85	6.28	Monoterpenoid
Levomenthol	8.76	4.50	7.34	Monoterpenoid
Terpinen-4-ol	8.93	1.87	3.05	Monoterpenoid
Methyl salicylate	9.05	3.52	5.74	Phenolic aromatic ester
Pulegone	9.68	2.06	3.36	Monoterpenoid
t-Butoxy-6-methylcyclohexene	9.82	3.16	5.15	Cyclic ether
linalyanthranilate	9.97	1.85	3.02	Monoterpenoid
4-Isopropyl-1,3-cyclohexanedione	10.04	2.70	4.40	diketone
Gamma-diosphenol	10.11	2.50	4.08	Monoterpenoid
2-[(E)-oct-2enyl] cyclopentan-1-one	10.26	1.92	3.13	ketone
Buchu camphor-diosphenol	10.56	4.29	6.99	Monoterpenoid
2,3a-Dimethylhexahydrobenzofuran-7a-ol	10.84	3.38	5.51	Monoterpenoid
4-Pentenoic acid, 2-methyl-4- nitro-, ethyl ester	11.23	3.34	5.45	Nito compound/ester
p-Mentha-8-thiol, trans	11.27	5.17	8.43	Monoterpenoid
2,3-dimethyl-2-(3-oxobutyl) cyclohexanone	13.46	1.78	2.90	ketone

RT: Retention time in minutes; A/H: area percent divided by height percent and RA%: relative abundance in percentage value.

### 3.2 Docking of metabolite from buchu essential oil with the target (PTP1B)

**Fig. 1:**
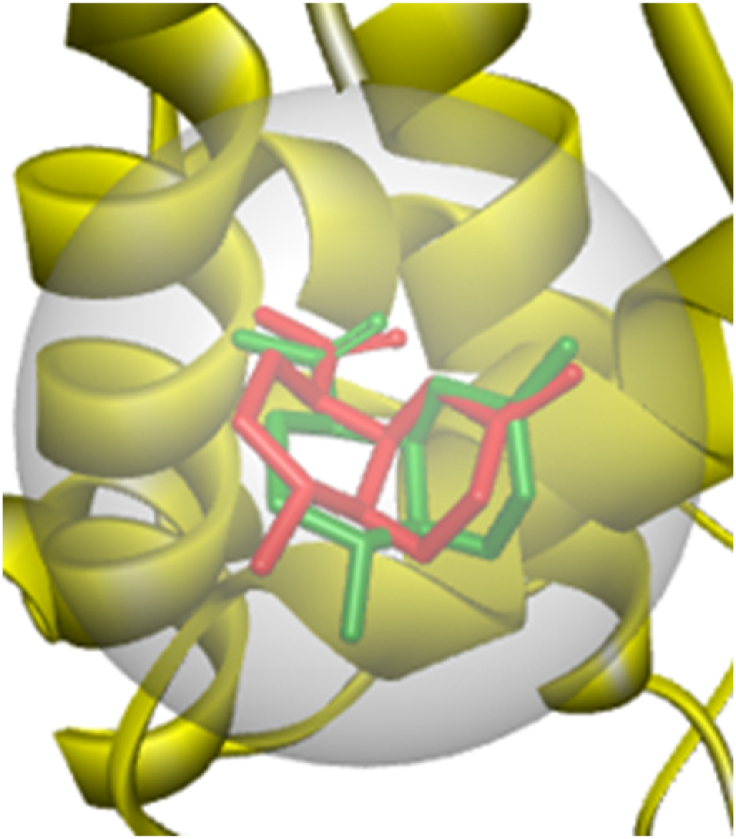
Redocked(red) and crystallographic poses(green) of PTP1B-co-crystallized ligand (amorphadiene) in the targets binding site.

Optimization of the docking protocol produces a redocked PTP1B-co-crystalized ligand (amorphadiene) pose that is almost superimposed on the crystallographic posed of the ligand as depicted in Fig. 1. Root mean square deviation (RMSD) of 0.98 Å was obtained between the two poses. This value fell within the acceptable threshold of 2 Å [27]. This high degree of superimposition validated the reliability of the docking protocol.

Molecular docking technique serves as a valuable tool for understanding ligand-target interactions. It positions a ligand within specific binding sites of target proteins to create stable complexes [28]. Generally, docking algorithms rated these interactions by negative binding scores. The magnitude of these scores describes how well a ligand fits within a protein's binding site, and strength of its interactions with the target [29]. Using the established protocol, the binding scores for the twenty-one metabolites from buchu and reference ursolic acid are presented in Supplementary Table S1. Linalylanthranilate emerged as the strongest binder, among the studied ligands, with a score of −5.2 kcal/mol, while p-menthon-8-thiol (trans) showed the weakest binding at −4.3 kcal/mol against PTP1B. These interactions suggest potential PTP1B-inhibitory properties of the ligands. While the reference standard ursolic acid (−6.7 kcal/mol) demonstrated superior binding, the ligands outperformed metformin, a known antidiabetic drug (though it acts through non-PTP1B mechanisms). The ligand-protein interactions in the study were stabilized through multiple mechanisms, including conventional hydrogen bonds, van der Waals forces, π-alkyl, π-sigma, and alkyl-alkyl interactions. Key amino acid residues involved in these interactions included TYR152, ALA189, ASN193, VAL287, CYS215, ARG221, AGNN66, ARG199, ASP181, TRP179, and PRO within the protein's binding pocket (Table S1, Figs. S1 and S2).

### 3.3 Binding free energy of top five metabolites from buchu essential oil

Molecular docking alone cannot definitively determine ligand-receptor binding affinity, as its scoring functions fail to account for the continuous motion of both ligand and protein atoms. Consequently, complementary methods like MD simulation are necessary to provide comprehensive insights into the energetics, stability, and dynamics of protein-ligand complexes. In this study, 150 ns MD simulation conducted on the complexes of top five metabolites (ligands) based on docking score: linanylantranilate (−5.2 kJ/mol), γ-diosphenol (−5.1 kJ/mol),4-isopropyl-1,3-cyclohexanedione (−5.0 kJ/mol), levomenthol (−4.9 kJ/mol) and p-menthol (−4.8 kJ/mol)(Supplementary Table S2) showed that van der Waals interaction (ΔG_vdw_) was the primary contributor to the total thermodynamic binding free energy (ΔG_bind_) across all investigated complexes (Table 2). This finding was supported by a strong positive Pearson's correlation coefficient (R = 0.903) between ΔG_vdw_ and ΔG_bind_ at P < 0.01 (Supplementary Table S3). The observation also aligns with Equation (1), which shows that more negative ΔG_gas_ lead to more negative ΔG_bind_ values, and Equation (3), which establishes the direct relationship between E_gas_ and E_vdw_. While ΔG_gas_ and ΔG_solv_ also showed positive correlations with ΔG_bind_, these correlations were less pronounced compared to ΔG_vdw_.

**Table 2:** Energy components of top five metabolites from buchu essential oil against PTP1B.

Component energies (kcal/mol)					
Systems	ΔE_vdW_	ΔE_elec_	ΔG_gas_	ΔG_solv_	ΔG_bind_
RS-PTP1B	−27.57 ± 5.08	−2.18 ± 5.16	−23.40 ± 6.71	7.50 ± 4.66	−15.89 ± 5.83
A-PTP1B	−24.48 ± 9.09	−5.55 ± 7.90	−30.03 ± 13.9	9.85 ± 7.44	−20.18 ± 8.48
B-PTP1B	−20.71 ± 2.97	−3.79 ± 3.78	−24.49 ± 4.84	8.00 ± 3.12	−16.49 ± 3.62
C-PTP1B	−17.91 ± 2.52	−5.36 ± 3.74	−23.26 ± 4.50	11.25 ± 4.04	−12.01 ± 3.00
D-PTP1B	−13.56 ± 7.44	−3.03 ± 4.30	−16.59 ± 9.84	4.94 ± 3.77	−11.65 ± 7.06
E-PTP1B	−9.74 ± 7.52	−3.52 ± 5.08	−13.26 ± 10.8	6.51 ± 5.86	−6.75 ± 5.89

RS = Reference compound (Usorlic acid); A = Linalylanthranilate; B = γ-diosphenol; C = 4-Isopropyl-1, 3-cyclohexanedione; D = Levomenthol; E = p-menthon-8-thiol; ΔE_vdW_ = van der Waals energy, ΔE_ele_ = electrostatic energy, ΔE_gas_ = gas-phase free energy, ΔG_solv_ = solvation free energy, ΔG_bind_ = total binding free energy.

MMGBSA calculated thermodynamic binding free energy (ΔG_bind_) of the top five ligands ranged from −20.18 ± 8.48 kcal/mol to −6.75 ± 5.89 kcal/mol as presented in Table 2. The MMGBSA method employed in molecular dynamic simulation has been reported to provide more accurate binding free energy calculations for protein-ligand interaction when compared to conventional docking scoring functions [30]. Furthermore, strength of ligand protein interaction has been shown to be directly proportional to the magnitude of ΔG_bind_ value [31]. Notably, linalylanthranilate (−20.18 ± 8.48 kcal/mol) and γ-diosphenol (−16.49 ± 3.62 kcal/mol) exhibited higher ΔG_bind_ values compared to the known PTP1B inhibitor, ursolic acid (−15.89 ± 5.83 kcal/mol), despite contrasting docking scores (Table 2 and Table S2). While ursolic acid demonstrated a higher negative ΔG_vdw_, the overall ΔG_gas_, which is determined by three components (E_int_, E_ele_, and E_vdw_) according to Equation (3), was more favorable for linalylanthranilate and diosphenol. The correlation between ΔG_gas_ and ΔG_bind_ is documented in Supplementary Table S2. The superior binding free energies of linalylanthranilate and diosphenol can be attributed to their enhanced internal energy and consequently more negative ΔG_gas_ values. Additionally, their higher positive ΔG_solv_ contributed to more favorable ΔG_bind_ values, indicating stronger binding affinity for PTP1B. These thermodynamic parameters suggest that linalylanthranilate and γ-diosphenol may exhibit superior PTP1B inhibitory activity compared to ursolic acid. Consequently, these compounds demonstrate the capacity to sustain insulin receptor phosphorylation kinetics, resulting in enhanced glucose transport efficacy, improved glycemic regulation, and augmented insulin sensitivity, thereby establishing their therapeutic potential as novel anti-T2DM interventions.

### 3.4 Post dynamic simulation analysis

Post dynamic simulation analyses provide detailed insights into protein-ligand interaction dynamics through evaluation of multiple thermodynamic parameters [32]. These analyses quantify conformational changes and binding-induced modifications through various parameters: root mean square deviation (RMSD) (assesses structural stability), root mean square fluctuation (RMSF) (measures residue flexibility), radius of gyration (ROG) (determines protein compactness), and solvent accessible surface area (SASA) (evaluates surface exposure). Additionally, hydrogen bond interaction analyses characterize the specificity and strength of molecular recognition. Together, these parameters provide comprehensive understanding of complex stability, protein conformational dynamics, and residue-specific responses to ligand binding.

#### 3.4.1 Root mean square deviation

**Fig. 2:**
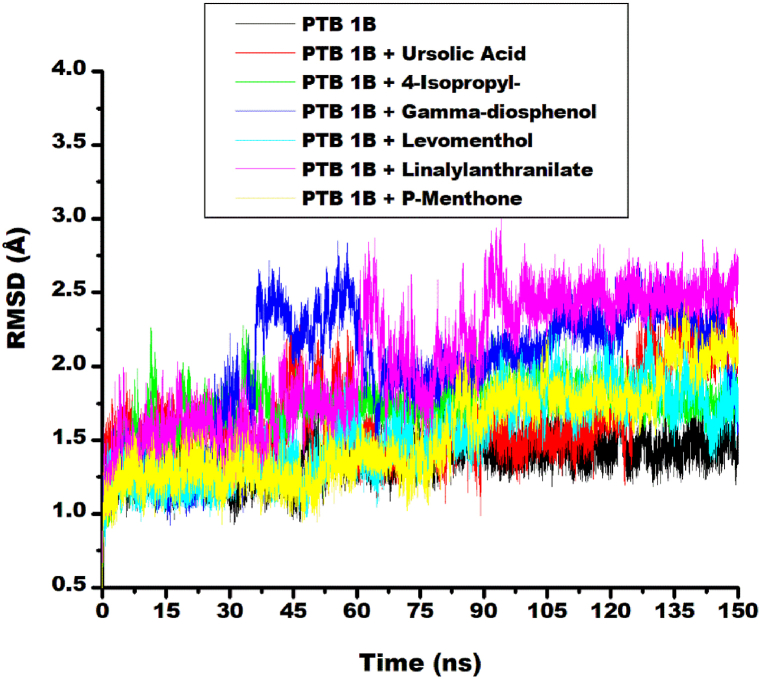
Comparative RMDS plots of alpha-carbon, ursolic acid and top five metabolite from buchu essential oil against PTP1B over 150 ns MD simulation.

In this current study, RMSD analysis revealed the initial equilibration of unbound protein and the buchu essential oil top five ligand-target complexes (i.e. metabolite bound to PTP1B) at approximately 7 ns. After the initial 7ns, notable conformational fluctuations were observed in linalylanthranilate-target and 4-isopropy-1,3-cyclohexanedione-target trajectories until 30 ns where γ-diosphenol-target complex joined in the fluctuations. The system converged briefly at 75 ns, with subsequent linalylanthranilate-target trajectory fluctuations and final convergence at 100 ns, after which the system maintain equilibrium throughout 150 ns simulation period (Fig. 2). Average root mean square deviation (RMSD) values of the ligand-target complexes exhibited varying fluctuations, with mean values ranging from 1.54 Å for Levomenthol to 2.05 Å for Linalylanthranilate (Table 3). These values were within the established threshold of ≤3 Å, indicating stable protein conformations [33]. Also, unbound PTP1B had a lower average RMSD (1.39 Å) compared to the ligand-target complexes, indicating better stability i.e. reduced structural stability was observed upon ligand binding. As reported in literature, RMSD analysis quantifies protein structural stability during molecular dynamics simulations [34] and its magnitude is inversely proportional to protein stability [35]. It also quantifies the structural deviation of the protein-ligand complex from its initial unbound (apo) conformation throughout the simulation trajectory [36].

**Table 3:** Post molecular dynamic simulation analysis parameters.

System	RMSD (Å)	RMSF(Å)	ROG (Å)	SASA (Å)	H-Bonds
PTP1B	1.39 ± 0.13	0.99 ± 0.61	19.08 ± 0.06	12915.97 ± 284.89	144.23 ± 7.76
RS-PTP1B	1.68 ± 0.28	1.09 ± 1.05	19.14 ± 0.09	12807.87 ± 284.65	146.98 ± 7.98

A-PTP1B	2.05 ± 0.43	1.21 ± 1.36	19.32 ± 0.11	13017.24 ± 391.08	145.24 ± 8.07
B-PTP1B	1.99 ± 0.41	1.16 ± 0.99	19.22 ± 0.07	12897.49 ± 311.02	141.91 ± 8.09
C-PTP1B	1.72 ± 0.16	1.06 ± 0.65	19.09 ± 0.07	12976.26 ± 294.27	144.97 ± 7.92
D-PTP1B	1.54 ± 0.27	1.09 ± 0.58	19.18 ± 0.11	13087.44 ± 333.93	144.59 ± 8.05
E-PTP1B	1.58 ± 0.34	1.14 ± 1.03	19.22 ± 0.13	12954.09 ± 287.95	144.36 ± 7.68

RS = Reference compound (Usorlic acid); A = Linalylanthranilate; B = γ-diosphenol; C = 4-Isopropyl-1, 3-cyclohexanedione; D = Levomenthol; E = p-menthon-8-thiol.

Higher RMSD values observed for the ligand-target complexes could be attributed to deviation in their trajectory compared to that of the apo protein during simulation. This swaying is more pronounced for Linalylanthranilate-target complex, hence the highest RMSD value (2.05 Å). Levomenthol (1.54 Å) and p-menthon-8-thiol, trans– (1.58 Å) complexes had RMSD value lesser than ursolic acid-target complex, hence better stability. This may be due to a similar fluctuation pattern with the apo protein for about 9 ns and only showed slight deviation afterward, maintaining equilibration with the apo-protein (Fig. 2). Generally, statistical analysis of post-dynamic parameters in this study (Supplementary Table S3), yielded an F-value of 3.201 (p > 0.05), indicating no statistically significant differences between the post-molecular dynamic parameters of unbound PTP1B and its complexes. Hence, combining this observation with RMSD values obtained, the overall stability of the protein and its complexes was not compromised during the simulation study. Similar observation was made in a study bioprospecting selected medicinal plants against the main protease and RNA-dependent RNA polymerase of SARS-CoV-2 [37]. In another study, chrysoeriol-7-glucuronide, ellagic acid hexoside, spiraeoside and sofosbuvir binding to RNA polymerase of rotavirus A showed increased RMSD value compared to apo protein despite their high Gibbs free binding energy [38].

#### 3.4.2 Radius of gyration (ROG)

**Fig. 3:**
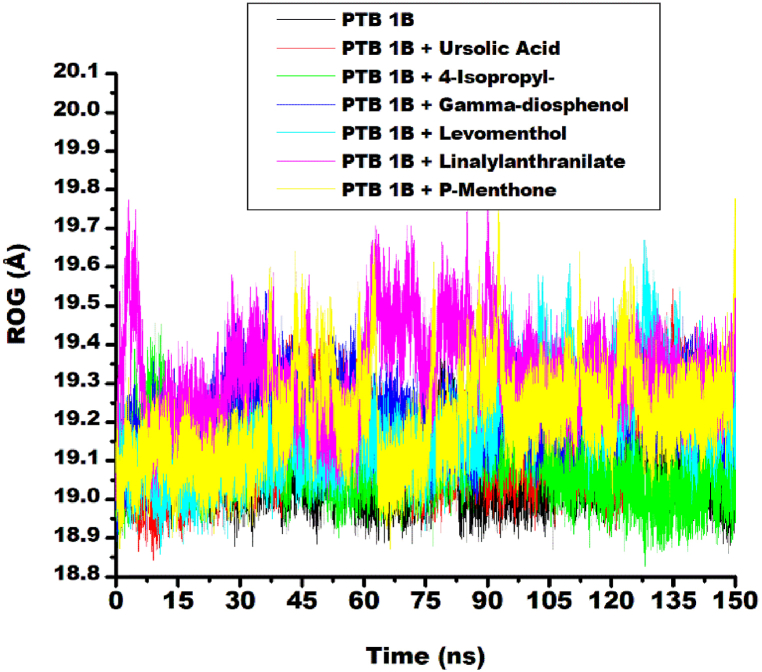
Comparative ROG plots of alpha-carbon, ursolic acid and top five metabolite from buchu essential oil against PTP1B over 150 ns MD simulation.

Another important dynamic parameter considered in the study is ROG. It quantitively measures the overall size, shape and compactness of a protein structure [39]. Typically, a stably folded protein with better compactness has low ROG value. While large ROG value is an indication of a more extended and flexible protein structure with reduced compactness [40]. In this study, similar ROG fluctuation patterns were observed for the ligand-target complexes and the unbound protein (target i.e. PTP1B) as depicted by Fig. 3. The systems were found to fluctuate between 18.9 Å to 19.7 Å. Linalylanthranilate-target complex trajectory (yellow spectrum in Fig. 3) demonstrated the highest fluctuation relative to the unbound protein (black spectrum), while 4-isopropy-1,3-cyclohexanedione-target complex (blue spectrum) demonstrated the lowest fluctuation relative to linalylanthranilate-target complex. The systems appear to converge at about 100 ns, except for linalylanthranilate-target complex trajectory that demonstrated minimal sway from the equilibration after convergence at 100 ns.

These fluctuations contributed to the average radius of gyration (ROG) values reported in the study, which ranged from 19.09 Å for 4-isopropy-1,3-cyclohexanedione-target complex to 19.32 Å for linalylanthranilate-target complex. The ROG for ligand-target complexes marginally exceeding the unbound PTP1B ROG value of 19.08 Å (Table 3). This slight elevation suggests a minimal reduction in protein compactness and folding upon ligand binding. The trend of this result was similar with the RMSD findings. The observed ROG values for the complexes were comparable to the reference compound, ursolic acid-target complex (19.14 Å) (Table 3) and may indicate better stability of the compounds.

The linalyanthranilate and γ-diosphenol complexes may be considered to have superior compactness going by their higher negative binding energy. Statistical analysis demonstrated a minimal variance of 0.005 between ROG values of unbound protein and that of the complexes, indicating no significant difference between ROG value of unbound PTP1B and ligand-target complexes. Therefore, ligand binding did not substantially alter the protein's global compactness. The submission in this study aligned with literature where marginal variation between ROG values of pinocembrin, chlorogenic,1,2,4,5-tetrazine-3,6-diamine, ursolic acid-PTP1B complexes and unbound PTP1B was suggested as a pointer to the compactness of the system [41].

#### 3.4.3 Root mean square fluctuation (RMSF)

**Fig. 4:**
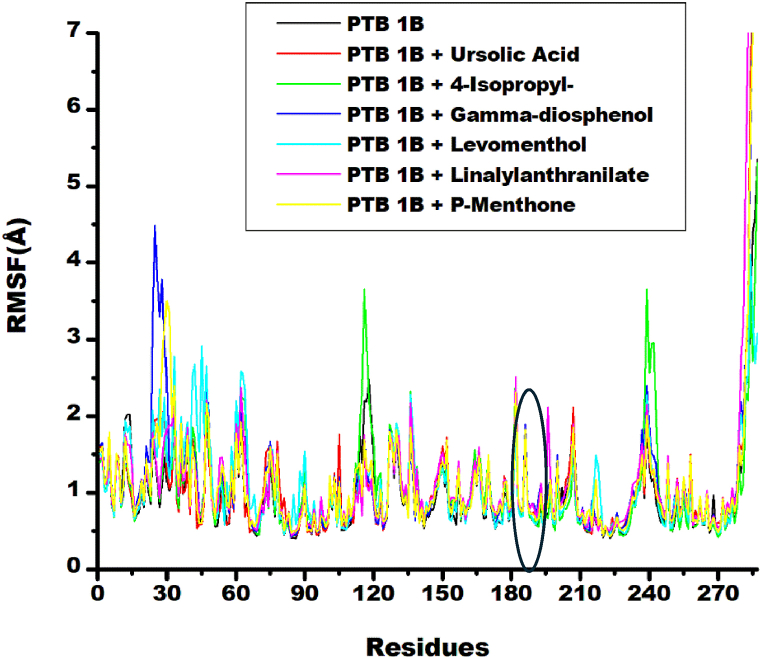
Comparative RMSF plots of alpha-carbon, ursolic acid and top five metabolite from buchu essential oil against PTP1B over 150 ns MD simulation.

The Root Mean Square Fluctuation (RMSF) analysis which evaluated the binding interactions between ligand and protein active site residues was also conducted in the study. RMSF quantifies the mean deviation of atomic positions from their average positions during molecular dynamics simulations, providing insights into protein structural flexibility and dynamics [34]. Higher RMSF values indicate increased flexibility, while lower values suggest structural rigidity [34]. The analysis revealed comparable fluctuation patterns between unbound PTP1B and ligand-target complexes, predominantly maintaining amplitudes below 3 Å (Fig. 4). Notable deviations were observed in specific residues: γ–diosphenol-PTP1B complex exhibited a 4.5 Å fluctuation at Ala27; Lianalylanthranlilate-PTP1B showed 3.5 Å and 2.4 Å fluctuations at Phe30 and Leu195, respectively; p-menthone-PTP1B demonstrated a 3.4 Å fluctuation at Phe30; metformin-PTP1B displayed a 3.5 Å fluctuation at Arg45; and 4-isopropy-1,3-cyclohexanedione exhibited 3.5 Å fluctuations at both Gly117 and Asp240 (Fig. 4). These fluctuations accounted for the variation in average RMSF values for the systems reported in Table 3, which were bounded by 1.06 ± 0.65 Å and 1.21 ± 1.36 Å. As reported in Table 3, unbound-PTP1B had a lower RMSF value (0.99 ± 0.61 Å) compared to that of ligand-target complexes, which were greater than 1 Å. This suggested enhanced flexibility of the protein upon ligand binding. RMSF values of 4-isopropy-1,3-cyclohexanedione (1.06 ± 0.65 Å) and Levomenthol (1.09 ± 0.58 Å) were marginally lower than that of the reference compound ursolic acid (1.09 ± 1.05 Å). Indicating the possibility of better sitting in the binding set of the protein target. Whereas RMSF values of linalylanthranilate (1.21 ± 1.36 Å) and Ƴ-Diosphenol (1.16 ± 0.99 Å), with higher binding energy ΔG, were marginally higher than that of the reference compound. This indicated enhanced flexibility of the protein on binding to these ligands which could better open the active site of this protein for more interaction. As previously mentioned for observed RMSD values, there is no statical difference between the RMSF values of the unbound-PTP1b and ligand-PTP1B complexes. The catalytic residue Ala189 demonstrated minimal fluctuation (0.5–1.8 Å) across all systems, indicating its active participation in both intra- and intermolecular binding interactions. Generally, ligand binding preserved the structural integrity of PTP1B. Similar RMSD values were reported in literature for ursolic acid-PTP1B (1.109 Å) and naringenin-PTP-1B (1.076 Å) [39].

#### 3.4.4 Solvent accessible surface area (SASA)

**Fig. 5:**
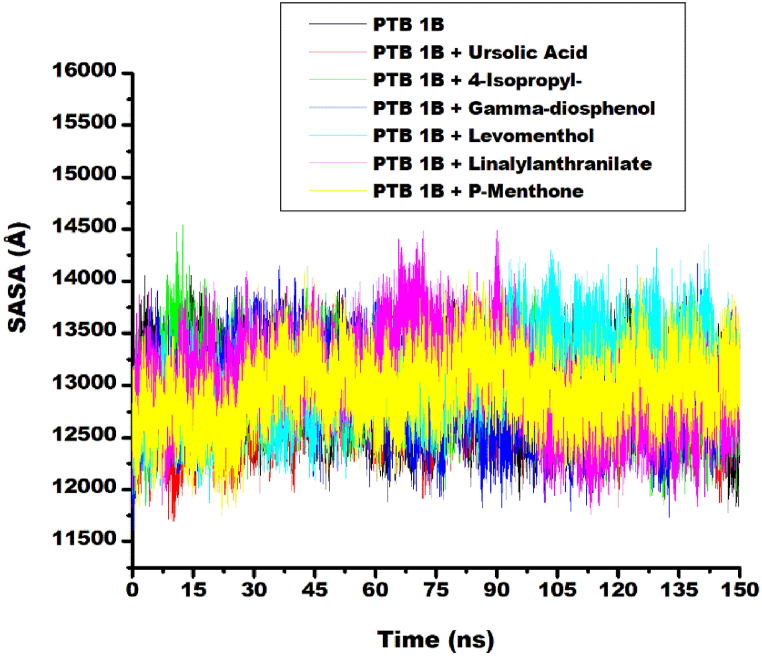
Comparative SASA plots of alpha-carbon, ursolic acid and top five metabolite from buchu essential oil against PTP1B over 150 ns MD simulation.

Another key indicator of protein conformational changes utilized to elucidate the dynamic behavior of the ligand-target complexes in this study is solvent-accessible surface area (SASA). It quantifies the hydrophilic and hydrophobic interactions between protein residues and solvent molecules. Lower SASA values in bound systems, compared to unbound proteins, indicate increased exposure of non-polar residues, resulting in enhanced complex stability. In this study, 4-isopropy-1,3-cyclohexanedione-target complex displayed the highest SASA fluctuation of 14500 Å at about 15 ns and Ursolic acid target complex showed the lowest SASA fluctuation at about 12 ns as shown in Fig. 5. Generally, there was a steady rise in SASA value uptil15 ns where the study systems displayed similar consistent fluctuation throughout the simulation period. This observation aligned with literature report for SASA trajectories of Diprotin A, ursolic acid and ranirestat complexed with PTP1B [39] and SASA plots for Abacopterin D, kaempferol 7-O- glucoside, Miconioside A, Quercetin 3-O- rhamnoside Quercitrin and Zafirlukast complexed with S-2MP [38]. Average SASA value for the unbound PTP1B (12915.97 ± 284.89 Å) was higher than that of Ursolic acid-target complex (12807.87 ± 284.65 Å) and γ-diosphenol-target complex (12897.49 ± 311.02 Å) (Table 3). This indicated increased exposure of hydrophobic non-polar residue to solvent, which increased the stability of the complex [42]. This result agrees with the high free binding energy ΔG_bind_ observed for Ursolic acid and γ-diosphenol. Furthermore, it was consistence with literature reports for Uroslic acid PTP1B and Diprotin A-PTP1B complexes [39]. Average RMSD values for Linalylanthranilate (13017.24 ± 391.08 Å), 4-isopropy-1,3-cyclohexanedione (12976.26 ± 294.27 Å), Levomenthol (13087.44 ± 333.93 Å) and p-menthon-8-thiol, trans – (12954.09 ± 287.95 Å) were marginally higher than that of the unbound PTP1B (Table 3). This implied marginal reduction in the exposure of hydrophobic target residue to solvent and consequent reduction in protein stability. However, one way ANOVA and Tukey HSD Post hoc test of post-dynamic parameters presented in Supplementary Tables S3 and S4 showed that no statistically significant differences between the post-molecular dynamic parameters of unbound PTP1B and its complexes. Hence, the overall stability of the system was not compromised through the simulation period.

#### 3.4.5 Intramolecular hydrogen bond and hydrogen bond distance

**Fig. 6:**
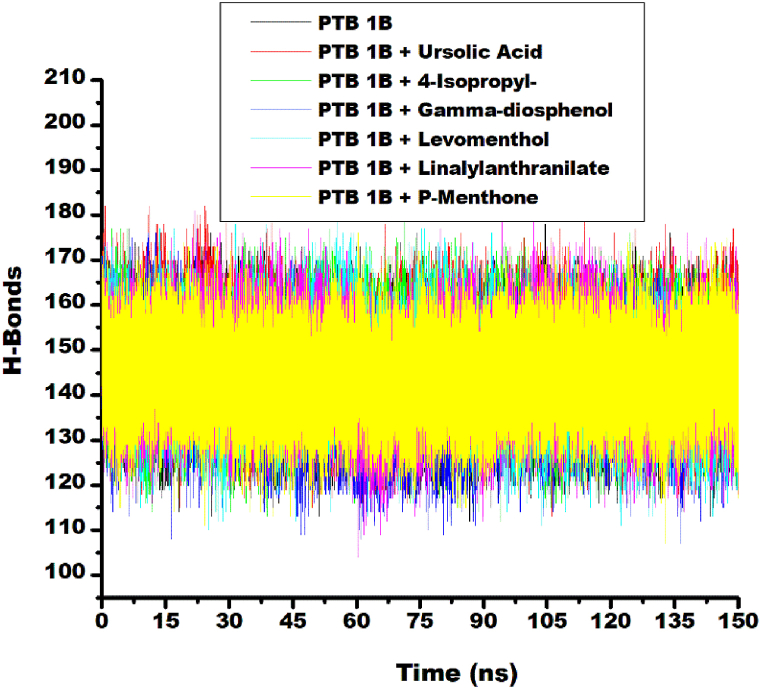
Comparative H-bond plots of alpha-carbon, ursolic acid and top five metabolite from buchu essential oil against PTP1B over 150 ns MD simulation.

**Fig. 7:**
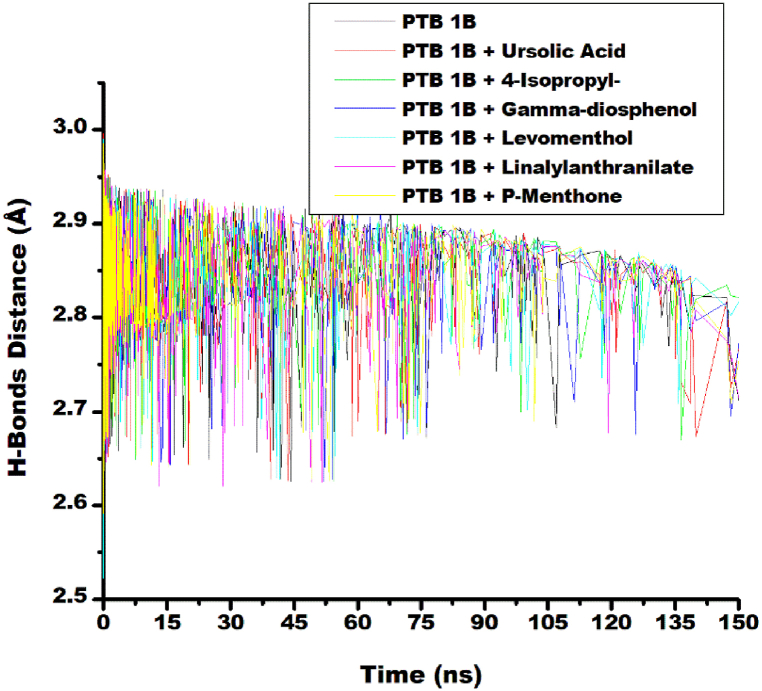
Comparative H-bond distance plots of alpha-carbon, ursolic acid and top five metabolite from buchu essential oil against PTP1B over 150 ns MD simulation.

Non-covalent interactions such as H-bonding and H-bond distances are crucial for the stabilization of protein–ligand complexes [43]. In this study, a stable and similar pattern of fluctuations in the number of H-bond interactions was observed for the ligand-target and unbound protein systems. This fluctuation was between 110 and 180 hydrogen bond (Fig. 6). This observation showed that the thermodynamic entropy of the protein target was not disrupted after binding with the top five ligands. An increase in the average number of hydrogen bonds was observed for the linalylanthranilate-PTP1B complex (145.24) compared to the unbound protein PTP1B (144.23). Similarly, increase number of H-bond was observed for levomenthol-PTP1B (144.59), 4-isopropy-1, 3-cyclohexanedione-PTP1B (144.97), p-menthon-8-thiol, trans - (144.36) and reference standard ursolic-acid complexes (146.98). However, relative to unbound protein, γ-diosphenol had lesser H-bond count (141.91) (Table 3). The increase in intramolecular hydrogen bonds because of ligands binding to PTP1B showed that the molecules occupy some intramolecular space within the structure of the protein to cause the increase. Similar result was reported in literature for the study phenolic as modulators of Penicillin-binding protein of *Staphylococcus aureus* [44]. Increased number of H-bond interactions observed for Linalylanthranilate-target and γ-diosphenol-target system compared to unbound-PTP1b is consistent with the high free binding energy ΔG_bind_ estimated for the complexes. The intermolecular-hydrogen bond distances of the ligand-target and unbound PTP1B initially fluctuated up to 2.95 Å and reduced towards 2.80 Å as the time progressed from 0 ns to 150 ns (Fig. 7). Average intramolecular-hydrogen-bond distance reported for all the systems in this study was 2.85 Å (Table 3). This suggests that the binding of PTP1B with the selected top five ligands and the reference standards did not disrupt the arrangement and the original geometry of PTP1B but rather caused more internal pull between the atoms and residues of PTP1B as the simulation progressed. This further emphasized the orderliness in the protein following the compounds binding at the active site, an observation that agrees with other thermodynamic metrics of this study.

The observed hydrogen bond distances demonstrate concordance with previous findings by Aribisala and Sabiu [45] who documented a progressive decrease in hydrogen bond lengths across both apo-protein and ligand-protein complexed systems, with a mean value of 2.85 Å. In another publication, comparable mean hydrogen bond lengths of 2.85 Å and 2.87 Å in systems targeting S-2 wild type and omicron spike protein, respectively, exhibiting a consistent reduction in hydrogen bond distance throughout the simulation period [46]. The hydrogen bond distances obtained in this study, further corroborates the thermodynamic stability of the selected top five ligands as established by their number of H-bond interaction and magnitude of their RMSD values.

#### 3.4.6 Bond analysis of interaction plots

Number, types and protein amino acid residue involved in the interactions between the top five metabolite and PTP1B are presented Table 4. Snapshots of the thermodynamic interaction in linanlyanthralinate-PTP1B, γ-diosphenol-PTP1B and Ursolic-acid-PTP1B complexes were shown in Fig. 8a, b and c respectively. It has been reported that the nature and the number of interactions existing between the ligand and target protein is critical to binding free energy value of the complex and elucidation of the degree of affinity [39]. linalyanthralinate-PTP1B complex, designated A-PTP1B in Table 4, had thirteen number of interactions comprising two hydrogen bonds, six van der Waals forces and five alkyl-alkyl interactions at the end of 150 ns MD simulation. These interactions translate into the high value ΔG_bind_ (−20.18 kcal/mol) reported for Linalyanthralinate-PTP1B complex in the study (Table 2), and it agrees with the prior observation that van der Waals interaction and number of hydrogen bonds played major role in the determination of the affinity of the ligands for the target protein.

**Table 4:** Identified interactions between the top five *A. betulina* metabolite and PTP1B.

Ligand-target	No. of int.	H-bonds	van der Waal forces	Others
RS-PTP1B	12	0	10 (ALA77, GLN78, ARG79, ARG199, GLU200, GLY202, LEU204, SER205, PRO206)	Alkyl-Alkyl (LYS237, LEU233)
A-PTP1B	13	2(GLY277, GLY277)	6(PRO180, ASN193, ARG199, GLU276, LYS279, ALA278)	Alkyl-Alkyl (ALA189, PHE196) π-Alkyl (LEU192, LEU195)
B-PTP1B	12	2(ASP245, ASP245)	6(MET74, GLU76, SER243, VAL244, ILE246, GLU252)	Alkyl-Alkyl (LEU234, ARG238, VAL249, LYS248)
C-PTP1B	9	2(ALA77, GLU79)	7(MET74, GLU75, THR230, LEU234, LYS248, VAL249, GLU252)	
D-PTP1B	9	0	5(GLN123, TRY124, PRO134, PHE135, GLU136	Alkyl-Alkyl (PRO89, CYS92, ALA122, MET133
E-PTP1B	0	0	0	0

RS = Reference compound (Usorlic acid); A = Linalylanthranilate; B = γ-diosphenol; C = 4-Isopropyl-1, 3-cyclohexanedione; D = Levomenthol; E = p-menthon-8-thiol.

**Fig. 8a:**
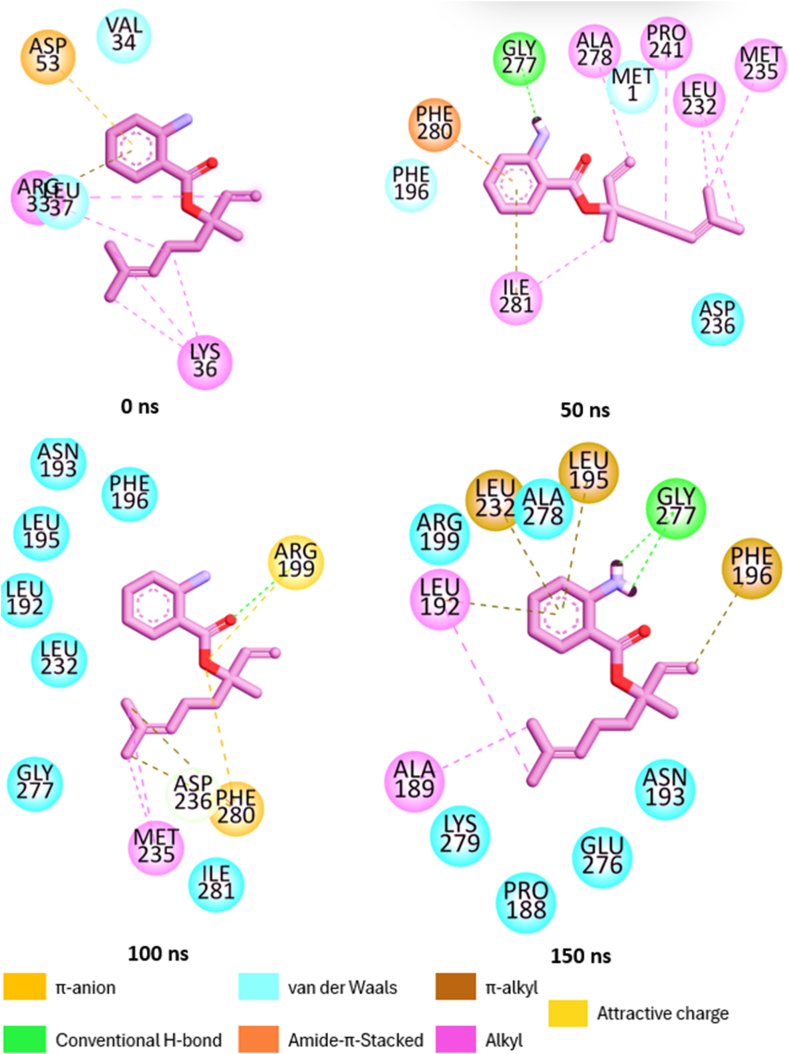
Snapshot of the post-dynamic interactions in the linaylantraninate-PTP1B system.

**Fig. 8b:**
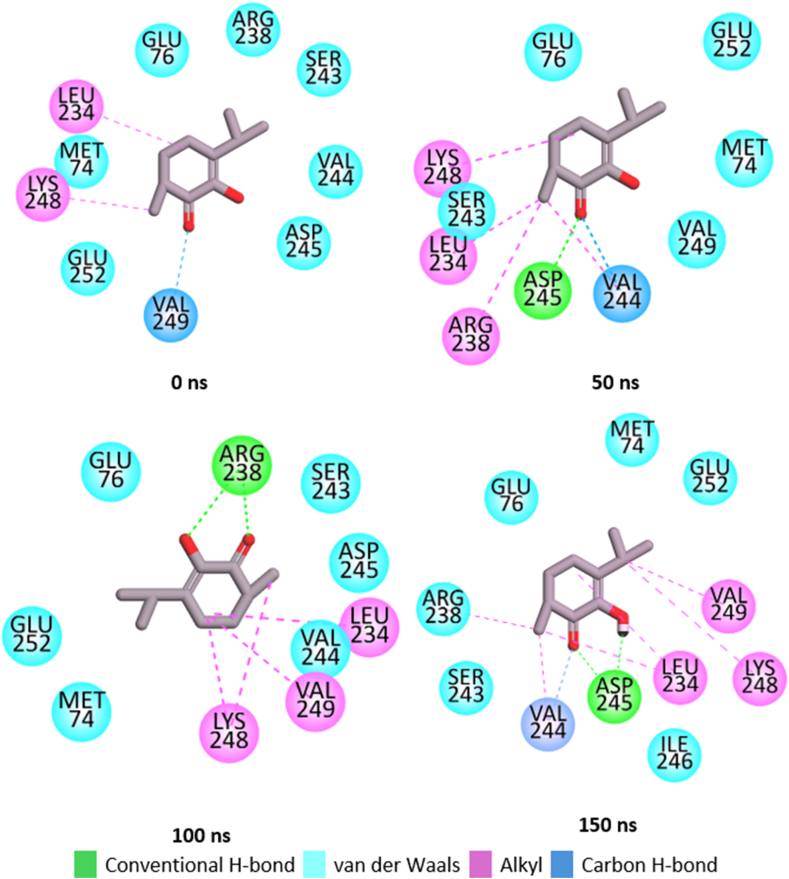
Snapshot of the post-dynamic interactions in the γ-diosphenol-PTP1B system.

**Fig. 8c:**
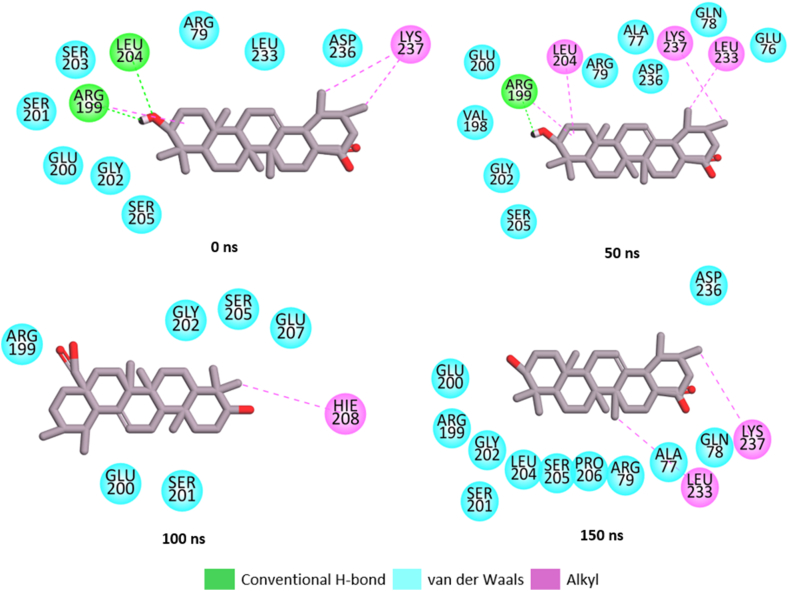
Snapshot of the post-dynamic interactions in the ursolic-acid-PTP1B system.

Ursolic acid (ten van der Waals forces, two alkyl-alkyl interactions) and γ–diosphenol (two hydrogen bond, six van der Waals forces and four alkyl-alkyl interactions) had twelve number of interactions which also translate into the high ΔG_bind_ observed for the two molecules. However, higher ΔG_bind_ observed for γ–diosphenol (−16.49 kcal/mol) compared to ursolic acid (−15.89 kcal/mol) could be attributed to the presence of hydrogen bond in γ–diosphenol which were not found in ursolic acid interaction with the target protein. Similar deduction can be made for the reported ΔG_bind_ for 4-isopropy-1, 3-cyclohexanedione-PTP1B and levomenthol which had nine interactions each at the end of 150 ns MD simulation, as it relates to the kind of interactions they had with the target PTP1B. p-menthon-8-thiol, trans with the lowest ΔG_bind_ displayed zero interaction with the PTP1B at the end of 150 ns MD simulation. This observation buttresses the claim that the number and type of interaction between the ligand and target protein was key to the determination of ΔG_bind_ reported after MD simulation. The result obtained in this study agreed with literature report of the interaction of ursolic acid, trans-cinnamic acid and Naringenin with PTP1B, where ursolic acid had lowest binding energy attributed to thirteen different interactions made with the target and Naringenin had higher binding energy attributed to 19 interactions it made with the target protein [39].

Molecular dynamic snapshots visualized through Discovery studio client, 2021as shown in Fig. 8a, Fig. 8ba, b and c for linalyanthranilate, γ-diosphenol and ursolic acid, respectively, further confirmed the crucial role of van der Waals interactions in this studied ligand-protein binding. As shown in Fig. 8a, the interactions analysis showed that linalyanthralilate binding to the target protein was facilitated through six van der Waals interactions with residues Pro188, Asn193, Arg199, Glu276, Ala278, and Lys279. Additionally, hydrogen bonding occurred with Gly277, while alkyl-alkyl interactions were established with Ala189, Leu192, Leu195, Phe196, and Leu232. In the case of γ-diosphenol, binding interactions included five van der Waals forces with Met74, Glu76, Ser243, Ile246, and Glu252, complemented by four alkyl interactions with Arg238, Val249, Leu234 and Cys248. One conventional hydrogen bonding with Asp245, and one carbon hydrogen bond with Val264 were also present (Fig. 8b). These findings align with previous studies by Abdulsalam et al. [47], which demonstrated plazomicin's role as an enzyme modulator in bacterial Gnaphalium affine, and Aribisala et al. [48], who investigated phenolic compounds as penicillin binding protein modulators. Furthermore, the reference compound ursolic acid also bonded with the investigated enzyme PTP1B via ten van der Waal interactions involving Ala77, Gln78, Arg79, Arg199, Glu200, Gly202, Leu204, Ser205, Pro206 and two strong alkyl-alkyl interactions involving Lys237 and Leu233. Similar result was obtained by Balogun et al. [39] where ursolic acid made thirteen interaction with PTP1B and nine of the interactions were van der Waals interactions. In the same vein, trans cinnamic acid was reported to made thirteen interactions with PTP1B and eight of the interactions were van der Waals interactions [33]. These buttresses the significant of van der Waals interactions in mediating the interactions between the ligands and protein used in this study.

### 3.5 Molecular orbital properties and electrostatic potential of top five ligands

**Fig. 9:**
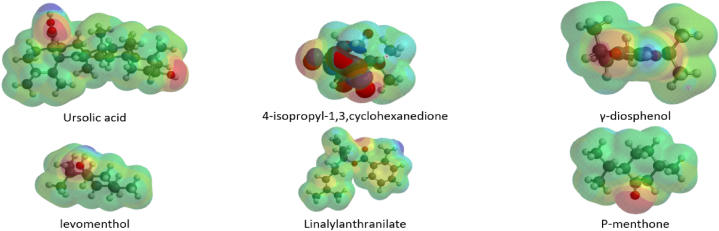
Electrostatic potential map of top five metabolites from buchu essential oil.

Density Functional Theory (DFT) calculations were done to support the finding on binding energy of top five ligand. It revealed molecular electrostatic potential (MEP) distributions crucial for protein-ligand interactions. The MEP maps displayed positive (blue) regions indicating electrophilic sites, neutral (green) regions with zero potential, and negative (red, orange, or yellow) regions denoting nucleophilic sites [14]. Linalylanthranilate exhibited extensive distribution of both positive and negative potential across its electrostatic map, correlating with its enhanced interaction profile in linalylanthranilate-PTP1B complexes. Similar distributions were observed in γ-diosphenol, ursolic acid, and 4-isopropyl-1,3-cyclohexanedione, contrasting with p-menthone's localized negative potential regions (Fig. 9). The findings in this study matched previous studies on distribution of positive and negative potential across *Capsicum Annuum* L metabolites with decreasing binding energies against DNA Gyrase B in the order: (1-Ethyloctyl) cyclohexane > Dibutyl phthalate > (1-Butylhexyl) cyclohexane [49].

**Fig. 10:**
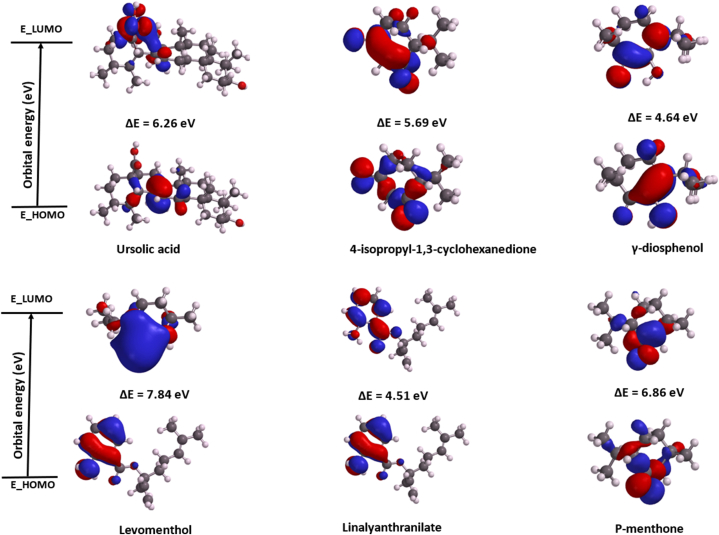
Frontier molecular orbital energy diagram for top five metabolite from buchu essential oil and ursolic acid.

Analysis of frontier molecular orbital energies, specifically HOMO (electron-donating capacity) and LUMO (electron-accepting capacity), provided critical insights into molecular medicinal properties [50]. The HOMO-LUMO energy gap (ΔE) serves as a key indicator of molecular stability and reactivity [51]. Linalylanthranilate demonstrated lower values for ΔE (4.51 eV), ionization energy (5.83 eV), and electron affinity (0.93 eV) compared to γ-diosphenol (ΔE = 4.64 eV, I = 6.00 eV, A = 1.36 eV) and ursolic acid (ΔE = 6.26 eV, I = 6.05 eV, A = −0.21 eV) (Table 5, Fig. 10). These parameters corresponded to their respective binding free energies (ΔG_bind_) of −20.18, −16.49, and −15.89 kcal/mol. The elevated energy gaps observed in levomenthol (7.84 eV) and p-menthone (6.86 eV) correlated with their reduced binding affinities compared to ursolic acid. The results obtained in this study is consistence with literature where secretin which demonstrated higher binding energy against trypsin-cleaved rotavirus A spike protein, had a lower energy gap compared to sacronoside A and 2SG, which demonstrated lower binding energies but had higher energy gaps [37].

**Table 5:** Density functional theory reactivity indexes for to five metabolites from buchu essential oil.

Properties	RS	A	B	C	D	E
E_LUMO (eV)	0.21	−0.93	−1.36	−0.94	2.01	−0.26
E_HOMO (eV)	−6.05	−5.44	−6.00	−6.63	−5.83	−7.12
ΔE (eV)	6.26	4.51	4.64	5.69	7.84	6.86
I (eV)	6.05	5.44	6.00	6.63	5.83	7.12
EA (eV)	−0.21	0.93	1.36	0.94	−2.01	0.26
η (eV)	0.61	0.04	−0.18	0.03	1.51	0.37
Ѕ (eV)^−1^	1.65	28.57	−5.56	33.33	0.66	2.70
μ (eV)	−2.92	−3.19	−3.68	−3.79	−1.91	−3.69
χ (eV)	2.92	3.19	3.68	3.79	1.91	3.69
ω (eV)	7.05	144.92	37.62	238.77	1.21	18.40

RS = Reference compound (Usorlic acid); A = Linalylanthranilate; B = γ-diosphenol; C = 4-Isopropyl-1, 3-cyclohexanedione; D = Levomenthol; E = p-menthon-8-thiol; I= Ionization energy; EA = Electron Affinity; η = Hardness; S = Softness; μ = Chemical potential; χ = Electronegativity index; ω = Electrophilicity Index.

Additional DFT-derived reactivity parameters, including hardness, softness, and electrophilicity index, evaluated results were presented in Table 5. Linalylanthranilate (hardness: 0.04 eV, softness: 28.57 eV) and 4-Isopropyl-1,3-cyclohexanedione (hardness: 0.03 eV, softness: 33.33 eV) exhibited values consistent with their superior binding energies [52]. The electrophilicity indices correlated with electronegativity scores, supporting the compounds' ability to act as an electrophile and its inhibitory potential. Notably, linalylanthranilate (144.92 eV) and γ-diosphenol (37.62 eV) with higher electrophilicity index demonstrated superior PTP1B inhibition compared to ursolic acid (7.05 eV), establishing their potential as antidiabetic therapeutic candidates. The result obtained in this study agrees with previous report on higher electrophilicity index of silicristin and ECMG compared to amoxicillin which supported the higher binding affinity of silicristin and ECMG compared to amoxicillin for penicillin binding protein (PBP) 2 × of Streptococcus pneumoniae [48].

### 3.6 Pharmacokinetic profile of the top five metabolites from buchu essential oil

The profiles of a compound in terms of bioavailability and toxicity can be predicted via ADME prediction. This is required when the compound is considered for drug development. One of the key rule employed in ADME predicted is Lipinski's rule of 5 (Ro5) which state that a prospective bioactive molecule can be considered for drug development provided its molecular weight is > 500 kDa, number of H donor is ≤ 5, number of H acceptor ≤10 and octanol-water partition coefficient is < 5 [41,53]. Based on the results of this study obtained from SwissAMDE and Protox II, all the top five metabolites passed Ro5 (Table 6). The implication is that, these five compounds have the potentials to be able to pass through the systemic circulation (unhindered) to elicit their pharmacological action as drug candidates [54]. Since the rate of absorption and amount of unchanged drug passing the blood is assessed by their bioavailability score (BS), this submission on Lipinski's rule was corroborated by their bioavailability score of 0.55 or 55 % and high GI absorption (Table 6). Generally, the minimally acceptable bioavailability limit for a drug candidate is 10 % [39,55], it then means that all the compounds have worthy profiles as probable candidates in the inhibition of PTP1B and management of T2DM. None of the top five metabolites are substrate to P-gp. Hence they cannot be actively efflux through biological membranes, for instance from the gastrointestinal wall to the lumen or from the brain [56]. The result also showed that the compounds studied are not inhibitors of cytochrome isoenzymes, CYP1A2, CYP2C19, CYP2D6, CYP2C9, CYP3A4 and CYP2E1. Cytochrome isoenzymes are key in drug metabolism. This signifies they are potentially safe and will not cause drug-drug toxicity. However, linalylanthranilate and γ-diosphenol showed activity against cytochrome CYP2C9 and CYP3A4, but the synthetic accessibility score reported for these metabolites was <5, suggesting they possess room for structural modification to improve potency and eliminate their activity against the cytochrome enzymes. They were further predicted to be inactive for hepatotoxicity, carcinogenicity, immunotoxicity, mutagenicity and cytotoxicity except for Linalylanthranilate that showed activity for hepatoxocity and mutagenicity. However, because of its synthetic accessibility value that is < 5, modification can be in cooperated into the structure to reduce its toxicity factor [57]. Based on the parameters obtained in this aspect of the study, linalyanthranilate and γ-diospheno from buchu essential oil (which possess higher ΔG_bind_ for PTP1B compared to that of the ursolic acid and have capability to be derivatized and synthesized for improved drugability and reduced toxicity) are potential candidates for PTP1B inhibition. Therefore, they have potential for enhancing insulin receptor sensitivity, improving glucose uptake, and providing other metabolic benefits generally necessary for T2DM intervention.

**Table 6:** Drug-likeness and ADMET properties of the top five metabolites from buchu essential oil.

Molecule	A	B	C	D	E
MW	273.37	168.23	154.21	156.27	154.25
Rot. bond	7	1	1	1	1
No. HBA	2	2	2	1	1
HBD	1	1	0	1	0
MR	84.48	49.37	43.66	49.23	48.27
LogP_(w/o)_	6	2	1.58	2.4	2.65
Ro5	0	0	0	0	0
BS	0.55	0.55	0.55	0.55	0.55
SA	2.91	3.07	2.13	2.63	2.42
GI abs.	High	High	High	High	High
BBB	No	Yes	Yes	Yes	Yes
Pgp-substrate	No	No	No	No	No
CYP1A2	No	No	No	No	No
CYP2C19	No	No	No	No	No
CYP2C9	Yes	Yes	No	Yes	No
CYP2D6	No	No	No	No	No
CYP3A4	Yes	No	No	No	No
CYP2E1	No	No	No	No	No
Hepatotoxicity	Yes	No	No	No	No
Carcinogenicity	No	No	No	No	No
Immunotoxicity	Yes	No	No	No	No
Mutagenicity	No	No	No	No	Yes
cytotoxicity	No	No	No	No	No

A = Linalylanthranilate; B = γ-diosphenol; C = 4-Isopropyl-1, 3-cyclohexanedione; D = Levomenthol; E = p-menthon-8-thiol, trans; SA = synthetic accessibility score; BS = Bioavailability score; Ro5 = Lipinski violation; GI = Gastrointestinal absorption.

## 4 Conclusion

The study revealed several key findings regarding binding capabilities of metabolites profiled in *Agathosma betulina* (buchu) essential oil with protein tyrosine phosphate 1B (PTP1B). The molecular docking score of the twenty-one metabolites ranged from −5.2 to −4.3 kcal/mol. Linalylanthranilate, γ-diosphenol, 4-Isopropyl-1, 3-cyclohexanedione, Levomenthol and p-menthon-8-thiol were selected as top five metabolites for molecular dynamic simulation based on their docking score. These exhibited binding free energies (ΔG_bind_) between −20.18 and −6.75 kcal/mol with linalylanthranilate (−20.18 kcal/mol) and γ-diosphenol (−16.49 kcal/mol) having superior PTP1B binding free energies than that of the reference compound ursolic acid (−15.98 kcal/mol). Post dynamic analysis of the interaction between the ligand and the target enzyme revealed that high ΔG_bind_ displayed by linalylanthranilate could be attributed to two H-bond it made with GLY277 amino acid residue of PTP1B and that of γ-diosphenol may be attributed to the two H-bond made with ASP245 amino acid residues, while ursolic acid, 4-Isopropyl-1, 3-cyclohexanedione, Levomenthol and p-menthon-8-thiol do not interact with the target via H-bonding. Furthermore, post dynamic analysis revealed that metabolite-PTP1B-complexes maintained stability throughout the 150 ns molecular dynamics simulation, with RMSD values ranging from 1.54 to 2.05 Å, below the 3 Å threshold. The radius of gyration (ROG) values (19.09–19.32 Å) were closely approximated to that of the unbound protein (19.08 Å). Additionally, RMSF values (1.09–1.21 Å) remained below 3 Å, indicating preserved protein folding integrity upon metabolite binding. Also, density functional theory (DFT) calculation showed that ΔG_bind_ values obtained for each complex in the study significantly correlate with the obtained DFT reactivity indices (LUMO-HOMO energy gap (ΔE), ionization potential (I), and electron affinity (A)) of corresponding compound. Finally, pharmacokinetic and ADMET predictions indicated favorable drug-like properties for the selected top five metabolites, with zero Lipinski violations, 55 % bioavailability scores, and high GI absorption. Synthetic accessibility values (2.13–3.07 Å) fell below the 5 Å threshold, suggesting potential for structural optimization. Therefore, the study suggests potential advantages of linalylanthranilate and γ-diosphenol as antidiabetic therapeutic candidates, however, further in vitro and in vivo validation studies are recommended and currently ongoing.

## CRediT authorship contribution statement

**Oluwaseye Adedirin:** Writing – review & editing, Writing – original draft, Visualization, Validation, Project administration, Methodology, Investigation, Formal analysis, Data curation, Conceptualization. **Rukayat A. Abdulsalam:** Methodology. **Khadeejah O. Nasir-Naeem:** Data curation. **Ayenitaju A. Oke:** Data curation. **Akolade O. Jubril:** Data curation, Conceptualization. **Saheed Sabiu:** Writing – review & editing, Visualization, Validation, Supervision, Software, Resources, Project administration, Methodology, Funding acquisition.

## Data availability statement

The data is contained within the article or supplementary material.

## Declaration of competing interest

The authors declare that they have no known competing financial interests or personal relationships that could have appeared to influence the work reported in this paper.
