# Supplementary material for: Density functional theory and molecular dynamics simulation-based bioprospection of Agathosma betulina essential oil metabolites against protein tyrosine phosphatase 1B for interventive antidiabetic therapy
Source: Heliyon. 2025 Jan 24;11(3):e42239. doi: 10.1016/j.heliyon.2025.e42239 (PMC11847251; doi:10.1016/j.heliyon.2025.e42239)
Supplement: Multimedia component 1 [file mmc1.docx]

Supplementary file


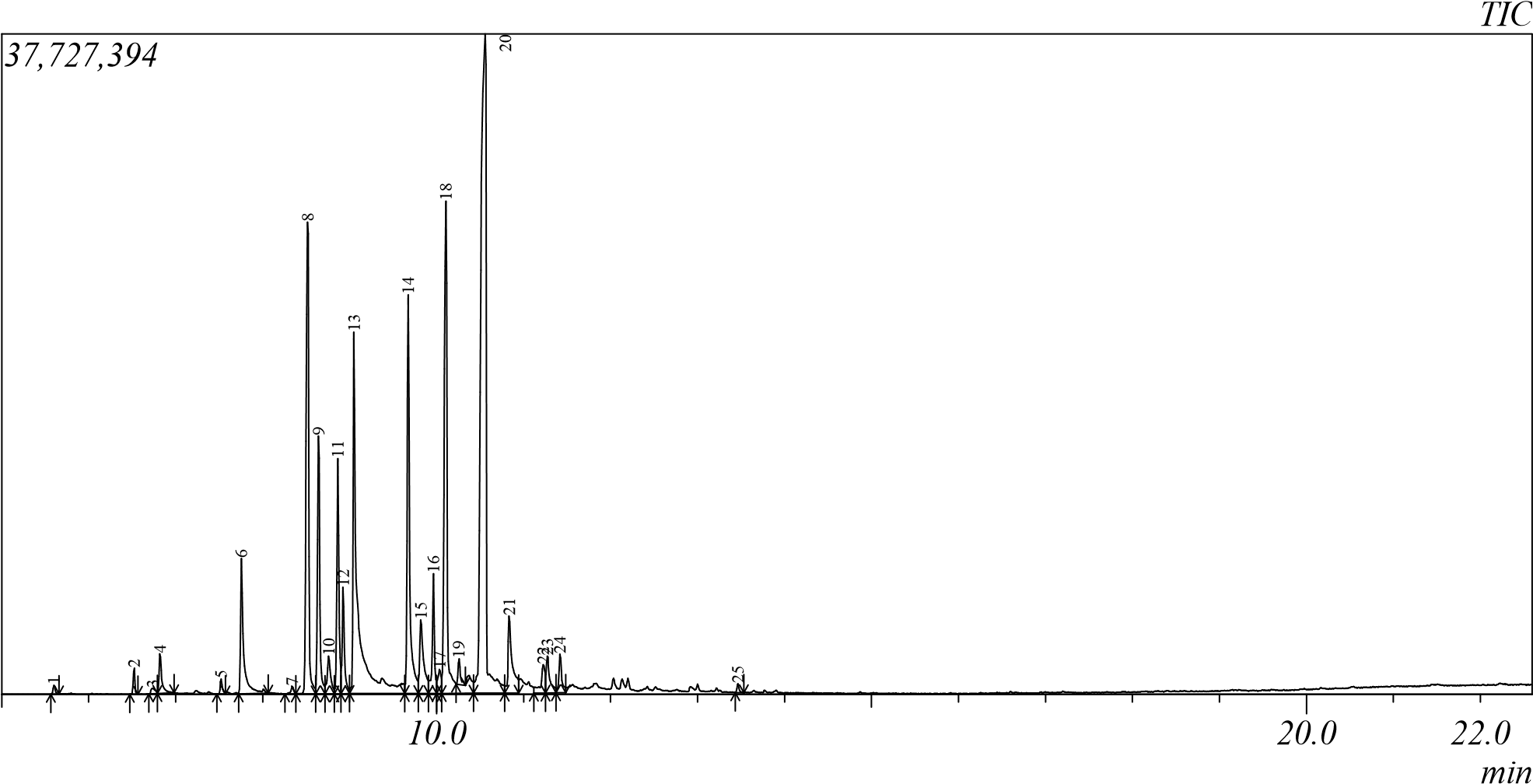


*Height %*

Figure S1: GCMS Chromatogram of buchu (*Agastosma betulina*) essential oil

Table S1: GCMS data for buchu (*Agasthosma betulina*) essential oil

| peak | RT | area | area% | height | height% | a/h | name |
| --- | --- | --- | --- | --- | --- | --- | --- |
| 1 | 5.602 | 920223 | 0.16 | 514434 | 0.24 | 1.79 | alpha-pinene] |
| 2 | 6.523 | 2123177 | 0.37 | 1483976 | 0.70 | 1.43 | cyclotetrasiloxane, octamethyl- |
| 3 | 6.736 | 1080386 | 0.19 | 300699 | 0.14 | 3.59 | beta-cymene |
| 4 | 6.820 | 5002587 | 0.87 | 2254051 | 1.07 | 2.22 | eucalyptol |
| 5 | 7.524 | 1526199 | 0.27 | 849480 | 0.40 | 1.80 | l-fenchone |
| 6 | 7.758 | 19060847 | 3.33 | 7731057 | 3.67 | 2.47 | (beta-linalool) |
| 7 | 8.343 | 895723 | 0.16 | 416089 | 0.20 | 2.15 | linalool,methyl ether |
| 8 | 8.520 | 56516853 | 9.88 | 26937682 | 12.78 | 2.10 | p-menthone |
| 9 | 8.644 | 25804969 | 4.51 | 14714494 | 6.98 | 1.75 | p-menthone |
| 10 | 8.760 | 5885569 | 1.03 | 2151728 | 1.02 | 2.74 | levomenthol |
| 11 | 8.866 | 23656278 | 4.14 | 13428511 | 6.37 | 1.76 | levomenthol |
| 12 | 8.926 | 11360904 | 1.99 | 6071408 | 2.88 | 1.87 | terpinen-4-ol |
| 13 | 9.051 | 73502125 | 12.85 | 20645471 | 9.80 | 3.52 | methyl salicylate |
| 14 | 9.675 | 46888857 | 8.20 | 22769320 | 10.80 | 2.06 | pulegone |
| 15 | 9.819 | 13249326 | 2.32 | 4196973 | 1.99 | 3.16 | ter-butoxy-6-methylcyclohexene |
| 16 | 9.965 | 12667801 | 2.21 | 6831924 | 3.24 | 1.85 | linaly anthranilate] |
| 17 | 10.035 | 3687023 | 0.64 | 1366122 | 0.65 | 2.70 | 4-isopropyl-1,3-cyclohexanedione |
| 18 | 10.108 | 71679760 | 12.53 | 28115875 | 13.34 | 2.50 | gamma-diosphenol |
| 19 | 10.257 | 2810983 | 0.49 | 1463981 | 0.69 | 1.92 | 2-[(e)-oct-2enyl] cyclopentan-1-one |
| 20 | 10.557 | 161365837 | 28.21 | 37652874 | 17.86 | 4.29 | buchu camphor-diosphenol |
| 21 | 10.836 | 14899835 | 2.60 | 4407739 | 2.09 | 3.38 | 2,3a-dimethylhexa  hydrobenzofuran-7a-ol |
| 22 | 11.227 | 5396049 | 0.94 | 1615250 | 0.77 | 3.34 | 4-pentenoic acid, 2-methyl-4- nitro-, ethyl ester |
| 23 | 11.274 | 5965371 | 1.04 | 2111583 | 1.00 | 2.83 | [p-mentha-8-thiol](https://pubchem.ncbi.nlm.nih.gov/compound/61982), trans |
| 24 | 11.419 | 5179337 | 0.91 | 217884 | 1.05 | 2.34 | [p-mentha-8-thiol](https://pubchem.ncbi.nlm.nih.gov/compound/61982), trans |
| 25 | 13.464 | 918918 | 0.16 | 515709 | 0.24 | 1.78 | 2,3-dimethyl-2-(3-oxobutyl)cyclohexanone |

Table S2: Docking score of metabolites from buchu essential oil against PTB1B

|  | Ligand | CID | Docking score (kcal/mol) | Amino acid |
| --- | --- | --- | --- | --- |
| ^1^ | ^b^Ursolic Acid | 64945 | −6.7 | `PHE196, ARG199, PHE280 |
| ^2^ | ^a^ Amorphadiene | 11052747 | −5.5 | ALA186, VAL287, PHE280 |
| 3 | Linalylanthranilate | 23535 | −5.2 | PRO180, ASP181, PHE182, ARG221, GLN266 |
| 4 | Terpinen-4-ol | 11230 | −5.2 | PHE196, ILE281 |
| 5 | Pulegone | 442495 | −5.1 | LEU192, ASN193, PHE280 |
| 6 | γ-Diosphenol | 108261 | −5.1 | ASP181 |
| 7 | Buchu camphor Diosphenol | 79023 | −5.1 | ALA189, PHE280 |
| 8 | 2,3-Dimethyl-2-(3-oxobutyl) cyclohexanone | 536789 | −5.0 | TRP179, GLY183, GLN266 |
| 9 | 4-Isopropyl-1,3-cyclohexanedione | 549661 | −5.0 | GLU76, ALA77 |
| 10 | 2-[(E)-oct-2enyl] cyclopentan-1-one | 76967591 | −4.9 | TYR152, ALA189, ASN193, VAL287 |
| 11 | Levomenthol | 16666 | −4.9 | ALA189, LEU192, ASNN193, PHE280 |
| 12 | 2,3a-dimethyl hexahydrobenzofuran-7a-ol | 579997 | −4.9 | TRP179 |
| 13 | Beta-Cymene | 7463 | −4.8 | LEU192, PHE196, PHE280 |
| 14 | Methyl salycilate | 23361 | −4.8 |  |
| 15 | ρ-Menthone | 26447 | −4.8 | 0 |
| 16 | L-Fenchone | 82229 | −4.7 | PHE196 |
| 17 | 4-pentenoic acid, 2-methyl-4- nitro-, ethyl ester | 549173 | −4.6 |  |
| 18 | Beta-Linalool | 6432254 | −4.5 | CYS215, ARG221, AGNN66 |
| 19 | 6-methyl-1-[(2-methylpro pan-2-yl)oxy] Cyclohexene | 574418 | −4.5 | ALA189, LEU 192, PHE 280 |
| 20 | Alpha-Pinene | 6654 | −4.5 | PHE196 |
| 21 | Eucalyptol | 2758 | −4.4 | PHE196 |
| 22 | Linalool,methyl ether | 108507 | −4.4 | TRY152, LEU 192, PHE280 |
| 23 | [p-Mentha-8-thiol, trans](https://pubchem.ncbi.nlm.nih.gov/compound/61982) | 61982 | −4.3 | PHE196, ARG199 |
| 24 | ^b^Metformin | 4091 | −3.9 | PHE196, ARG199 |

^a^Native ligand; ^b^ reference standard; CID: PubChem compound identity number


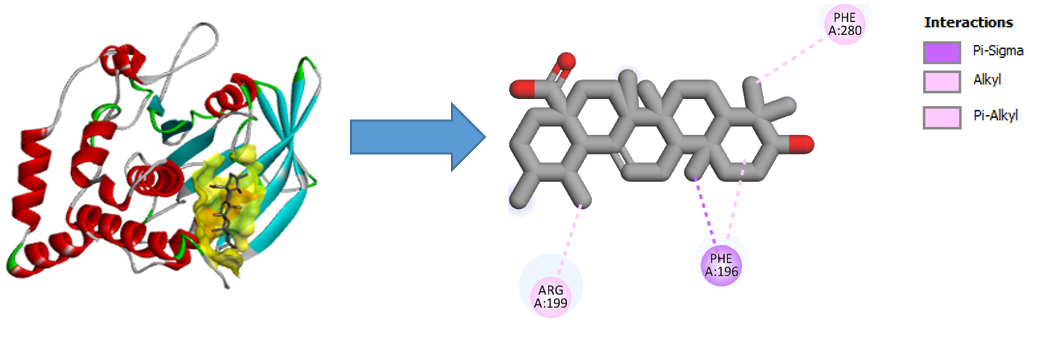


Figure S2. Binding interaction between Ursolic acid and PTP1B


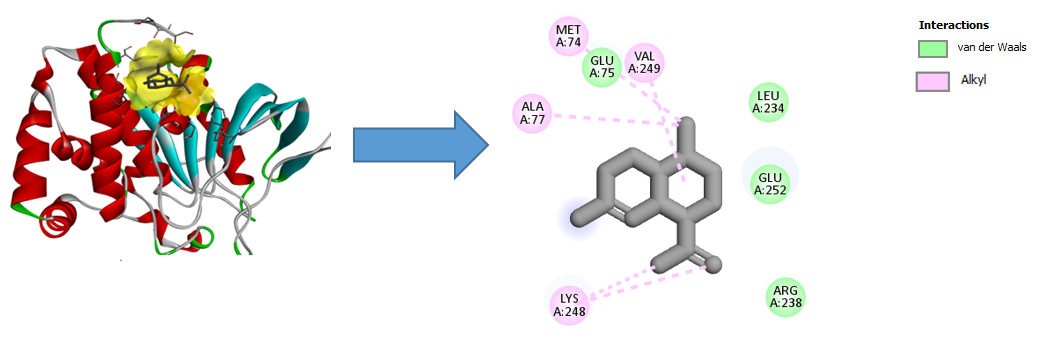


Figure S3. Binding Interaction between Amorphadiene and PTP1B


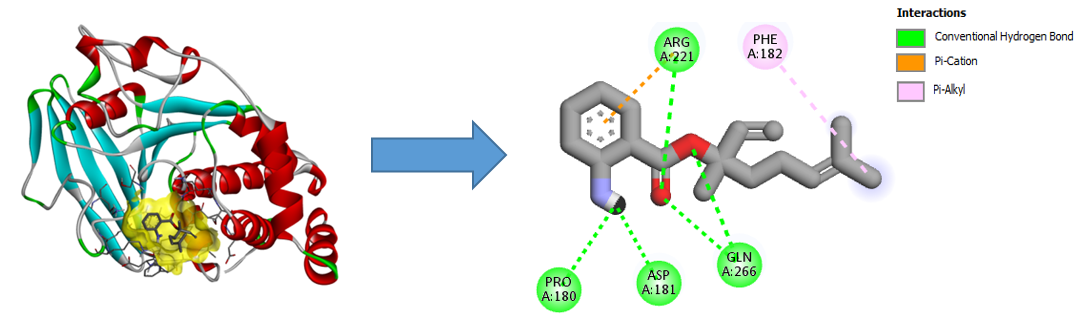


Figure S4. Binding Interaction between Linalylanthranilate and PTP1B (PDB: 6w30)


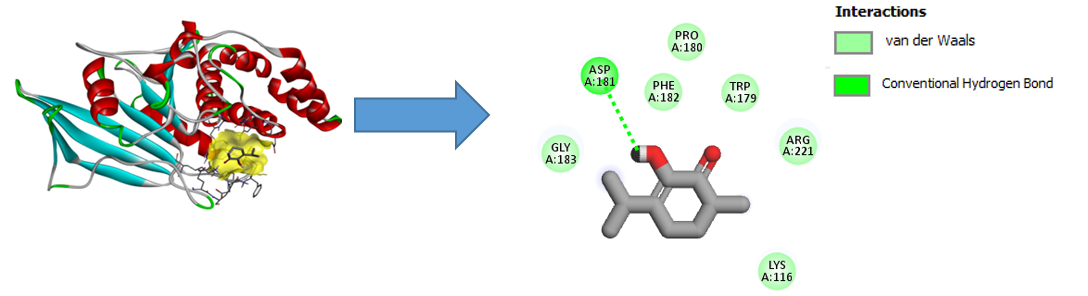


Figure S5. Binding Interaction between γ-diosphenol and PTP1B (PDB: 6w30)

Table S3. Pearson’s correlation analysis for top five metabolites thermodynamic parameters

|  | | ΔG | ΔE_vdW_ | ΔE_elec_ | ΔG_gas_ | ΔG_solv_ |
| --- | --- | --- | --- | --- | --- | --- |
| ΔG | Pearson Correlation | 1 |  |  |  |  |
|  | Sig. (2-tailed) |  |  |  |  |  |
| ΔE_vdW_ | Pearson Correlation | 0.903^**^ | 1 |  |  |  |
|  | Sig. (2-tailed) | 0.005 |  |  |  |  |
| ΔE_elec_ | Pearson Correlation | −0.514 | −0.705 | 1 |  |  |
|  | Sig. (2-tailed) | 0.238 | 0.077 |  |  |  |
| ΔG_gas_ | Pearson Correlation | 0.507 | 0.268 | 0.451 | 1 |  |
|  | Sig. (2-tailed) | 0.246 | 0.562 | 0.310 |  |  |
| ΔG_solv_ | Pearson Correlation | 0.431 | 0.599 | −0.981^**^ | −0.557 | 1 |
|  | Sig. (2-tailed) | 0.335 | 0.156 | 0.000 | 0.194 |  |

Table S4. ANOVA of Post molecular dynamic simulation parameters

|  | Sum of Squares | df | Mean Square | F | Sig. |
| --- | --- | --- | --- | --- | --- |
| Between Groups | 12.236 | 6 | 2.039 | 3.201 | .016 |
| Within Groups | 17.840 | 28 | 0.637 |  |  |
| Total | 30.076 | 34 |  |  |  |

Table S5. Tukey HSD Post hoc test for post molecular dynamic simulation parameters

| (I) GROUPS | (J) GROUPS | Mean Difference (I-J) | Std. Error | Sig. |
| --- | --- | --- | --- | --- |
|  |  |  |  |  |
| PTP1B | Usorlic acid | −0.748398 | 0.504 | 0.752 |
|  | Linalyanthranilate | −2.143358^*^ | 0.504 | 0.004 |
|  | Gama diosphenol | −1.063446 | 0.504 | 0.376 |
|  | Isopropyl | −0.741092 | 0.504 | 0.761 |
|  | levomenthol | −1.092702 | 0.504 | 0.345 |
|  | menthone | −1.037058 | 0.504 | 0.405 |
| Usorlic acid | PTP1B | 0.748398 | 0.504 | 0.752 |
|  | Linalyanthranilate | −1.394960 | 0.504 | 0.119 |
|  | Gama diosphenol | −0.315047 | 0.504 | 0.995 |
|  | Isopropyl | 0.007306 | 0.504 | 1.000 |
|  | levomenthol | −0.344303 | 0.504 | 0.993 |
|  | menthone | −0.288660 | 0.504 | 0.997 |
